# The role of AdhE on ethanol tolerance and production in *Clostridium thermocellum*

**DOI:** 10.1016/j.jbc.2024.107559

**Published:** 2024-07-11

**Authors:** Angel Pech-Canul, Sarah K. Hammer, Samantha J. Ziegler, Isaiah D. Richardson, Bishal D. Sharma, Marybeth I. Maloney, Yannick J. Bomble, Lee R. Lynd, Daniel G. Olson

**Affiliations:** 1 Thayer School of Engineering, Dartmouth College, Hanover, New Hampshire, USA; 2 Center for Bioenergy Innovation, Oak Ridge National Laboratory, Oak Ridge, Tennessee, USA; 3 Biosciences Center, National Renewable Energy Laboratory, Golden, Colorado, USA

**Keywords:** alcohol dehydrogenase, acetaldehyde dehydrogenase, ethanol tolerance, ethanol production, cellulosic biofuels, Hungateiclostridium, Ruminiclostridium, *Acetivibrio thermocellus*

## Abstract

Many anaerobic microorganisms use the bifunctional aldehyde and alcohol dehydrogenase enzyme, AdhE, to produce ethanol. One such organism is *Clostridium thermocellum*, which is of interest for cellulosic biofuel production. In the course of engineering this organism for improved ethanol tolerance and production, we observed that AdhE was a frequent target of mutations. Here, we characterized those mutations to understand their effects on enzymatic activity, as well ethanol tolerance and product formation in the organism. We found that there is a strong correlation between NADH-linked alcohol dehydrogenase (ADH) activity and ethanol tolerance. Mutations that decrease NADH-linked ADH activity increase ethanol tolerance; correspondingly, mutations that increase NADH-linked ADH activity decrease ethanol tolerance. We also found that the magnitude of ADH activity did not play a significant role in determining ethanol titer. Increasing ADH activity had no effect on ethanol titer. Reducing ADH activity had indeterminate effects on ethanol titer, sometimes increasing and sometimes decreasing it. Finally, this study shows that the cofactor specificity of ADH activity was found to be the primary factor affecting ethanol yield. We expect that these results will inform efforts to use AdhE enzymes in metabolic engineering approaches.

Cellulose is an abundant natural polymer that could be used to produce low-cost renewable fuels and chemicals. *Clostridium thermocellum* (also known as *Hungateiclostridium thermocellum*, *Ruminiclostridium thermocellum*, and *Acetivibrio thermocellus* (1)) is one of the best cellulose-fermenting organisms known (2, 3). However, wild type (WT) strains are sensitive to inhibition by potential biofuels such as ethanol, and this lack of tolerance limits product titer. The reasons for this sensitivity, however, are poorly understood.

Early work on microbial ethanol tolerance focused on the effects of membrane disruption (4, 5, 6). However in *C. thermocellum* (and many other anaerobic bacteria), growth is inhibited at ethanol concentrations that are thought to be too low to affect membrane fluidity (7). Furthermore, direct measurement of membrane energization shows no effect of ethanol on membrane energization, even at concentrations up to 40 g/L (enough to completely inhibit growth in WT *C. thermocellum*) (8). Finally, adaptive evolution studies in the presence of added ethanol have consistently shown mutations in the metabolic enzymes (9, 10, 11) and not in enzymes associated with membrane biosynthesis.

In *C. thermocellum*, the bifunctional metabolic enzyme AdhE mediates the conversion of acetyl-CoA to acetaldehyde (*i.e.* acetaldehyde dehydrogenase (ALDH) activity) and the subsequent conversion of acetaldehyde to ethanol (*i.e.* alcohol dehydrogenase (ADH) activity) *via* two separate domains. Spontaneous mutations in AdhE have been observed in response to gene deletions, adaptive evolution for improved growth, and adaptive evolution for improved ethanol tolerance (Fig. 1). One of the earliest mutations observed in AdhE was the AdhE^∗^ mutation (consisting of two mutations: P704L and H734R). This mutation originated in a strain adapted for increased ethanol tolerance after chemical mutagenesis (12); however, the mutations were only discovered after whole-genome resequencing of the strain (10). Reintroduction of these mutations into WT *C. thermocellum* was able to restore most of the ethanol tolerance phenotype.

**Figure 1:**
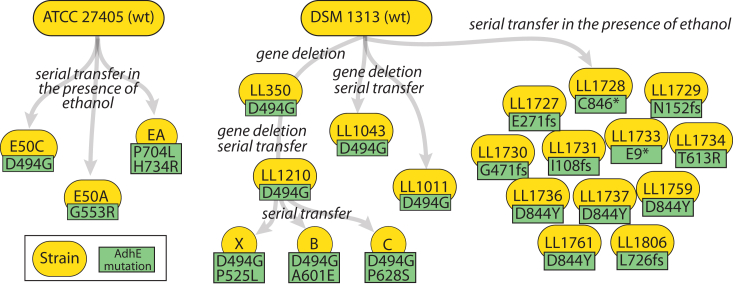
**Lineage of AdhE mutations in strains engineered for increased ethanol production or adapted for increased growth rate in the presence of added ethanol**. *Yellow circles* represent strains. *Green boxes* represent AdhE mutations. Adapted from (49).

Mutations in other strains of *C. thermocellum* adapted for increased ethanol tolerance include G553R (11), D844Y (9), and T613R (9), as well as a large number of frameshift and truncation mutants that would be expected to completely eliminate activity (9) (Fig. 1).

Another mutation, D494G, has occurred independently five times. It was first identified in a strain of *C. thermocellum* adapted for growth in the presence of added ethanol (Strain E50C) (11). It was subsequently identified in a strain of *C. thermocellum* engineered for increased ethanol production by disruption of hydrogen production (13), and two separate lineages of *C. thermocellum* engineered for increased ethanol production by elimination of lactate and acetate production (14, 15). Finally, it was observed in a strain of *C. thermocellum* adapted for butanol tolerance (16). Biochemical characterization of this mutation showed that it increased NADPH-linked ADH activity (17). Reintroducing this mutation into *C. thermocellum* has been shown to increase the ethanol yield (15, 18).

In a strain of *C. thermocellum* engineered for increased ethanol production by deleting pathways for hydrogen, formate, lactate, and acetate production (19), the D494G mutation was already present (inherited from the *hydG* deletion strain ancestor (13)). Additional serial transfers of this strain in the presence of high substrate concentrations (50 g/L cellobiose) resulted in the appearance of three additional mutations: P525L, A601E, and P628S.

Many of the mutations that improve ethanol tolerance in *C. thermocellum* decrease ethanol production (9) and, thus, we have observed a tradeoff between ethanol tolerance and ethanol titer in strains developed thus far. The goal of this work is to investigate the roles of *adhE* point mutations on ethanol tolerance in *C. thermocellum* in order to inform design of improved biofuel production pathways that avoid this tradeoff. Specifically, we aim to better understand why AdhE is such a frequent target for mutations, how ethanol tolerance affects ethanol production, and the underlying biochemical basis for these phenotypes. Previously, we have observed that *adhE* gene is a hotspot for mutations in strains of *C. thermocellum* engineered for increased ethanol tolerance or production (9, 20). However, the diverse genetic backgrounds of these strains made it difficult to isolate the effects of *adhE*. To directly compare the effects of *adhE* in a consistent genetic background, we cloned and expressed *adhE* mutants from a replicating plasmid (pDGO143 (18)) in a strain where the WT *adhE* gene had been deleted (LL1111 (21)).

## Results

### Effect of AdhE mutations on ethanol tolerance

First, we measured the effect of AdhE mutations on ethanol tolerance. We started with a strain of *C. thermocellum* where the *adhE* gene had been deleted (LL1111) (21). This strain converts cellobiose to lactate and acetate, but has lost the ability to produce ethanol. In this background, we expressed various *adhE* mutants (Table 1) from a replicating plasmid, pDGO143 (18) under control of the strong constitutive Clo1313_2638 promoter (22), which can functionally complement the *adhE* deletion (18, 22).

**Table 1:** AdhE mutations

AdhE mutation^a^	Origin of mutation	Cth plasmid ID^b^	Cth strain ID^c^	Eco plasmid ID^d^	Plasmid ID^e^
E328K	Unintentional error, see E328K D844Y	(not tested)	(not tested)	AP2-25	pAP15
E328K D844Y	The E328K mutation was unintentionally introduced when cloning the D844Y mutation.	(not tested)	(not tested)	AP2-40	pAP19
D494G	It has appeared several times, both in strains adapted for increased ethanol tolerance, and in strains engineered for increased ethanol yield (15, 17).	pSKH008	LL1823	AP2-24	pAP5
D494G P525L	Adaptation of high yielding strain (LL1210) to high substrate concentrations by serial transfer in the presence of 50 g/L cellobiose for ∼2000 generations (18).	pSKH009	LL1829	AP2-23	pAP6
D494G A558E	The A558E mutation was unintentionally introduced when cloning the D494G mutation.	(not tested)	(not tested)	AP2-05	pAP18
P525L	Same conditions as D494G P525L mutation	pSKH004	LL1820	AP2-10	pAP10
G552D	Re-creation of a mutation found in *Thermoanaerobacterium saccharolyticum* AdhE (50)	(not tested)	(not tested)	AP2-07	pAP23
G553R	Adaptation to high ethanol tolerance (11)	pSKH002	LL1818	AP2-08	pAP8
A601E	Same conditions as D494G P525L mutation	pSKH001	LL1824	AP2-04	pAP13
P628S	Same conditions as D494G P525L mutation	pSKH005	LL1821	AP2-11	pAP11
P704L	See P704L H734R	pSKH006	LL1822	AP2-06	pAP12
H734R	See P704L H734R	pSKH003	LL1819	AP2-09	pAP9
P704L H734R	Adaptation to high ethanol tolerance (10)	pSKH010	LL1830	AP1-18	pAP7
D844Y	Adaptation to high ethanol tolerance (9)	(not tested)	(not tested)	AP2-21	pAP17

a Sorted by mutation position.

b *C. thermocellum* expression plasmid.

c *C. thermocellum* strain containing expression plasmid.

d *Escherichia coli* protein expression plasmid.

e For protein expression in *E. coli* used for *in vitro* protein characterization.

**Figure 2:**
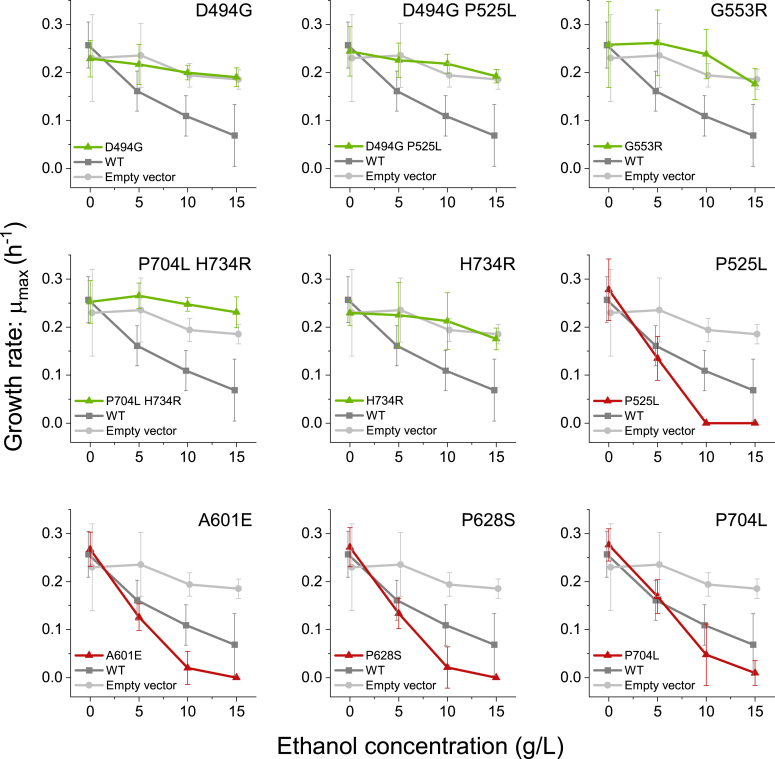
**Growth rate as a function of added ethanol concentration for AdhE mutants expressed from a replicating plasmid in an *adhE* deletion strain (LL1111).** In each panel, the WT AdhE and empty-vector controls are included for reference (*dark gray* and *light gray*, respectively), also expressed in the *adhE* deletion strain. (Note that the data labeled WT represents the WT *adhE* gene expressed in the LL1111 *adhE* deletion strain). Mutants are further color coded to indicate groups of ethanol tolerance. *Green* indicates increased tolerance relative to the WT AdhE, *red* indicates decreased ethanol tolerance relative to the WT AdhE. Within each group mutants are ordered by amino acid position. Error bars represent one standard deviation, n ≥ 4 biological replicates. Within each plot, the WT and empty vector control data points are slightly offset along the *x*-axis (ethanol concentration) to avoid obscuring the mutant data.

Strains expressing different mutant *adhE* genes were grown in the presence of different concentrations of ethanol from 0 to 15 g/L, and the maximum specific growth rate (μ_max_) was measured (Fig. 2). We observed that the effect of the mutant *adhE* genes could be divided into two groups. One group (Fig. 2 - green lines) showed ethanol tolerance that was increased relative to the WT control, and similar to the empty vector control. Another group (Fig. 2 - red lines) showed ethanol tolerance that was decreased relative to the strain expressing WT AdhE. Expression of WT *adhE* in an *adhE* deletion strain (strain LL1111) resulted in similar ethanol tolerance to the WT strain (Fig. S4).

### Effect of AdhE mutations on ethanol production

**Figure 3:**
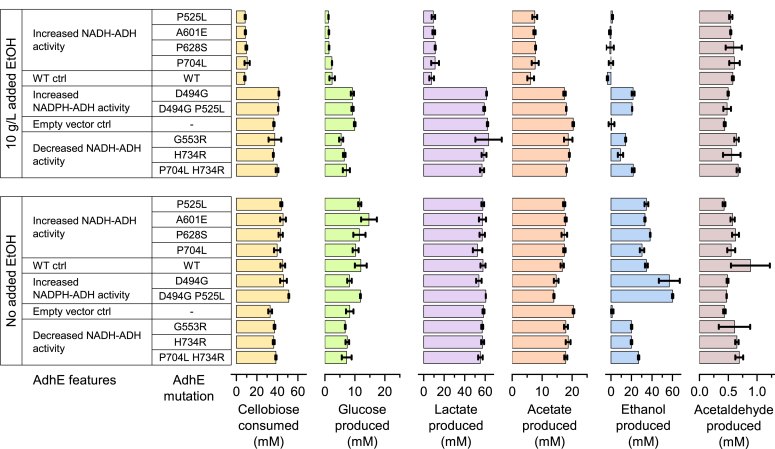
**Fermentation products for various *adhE* mutants expressed in *Clostridium. thermocellum adhE* deletion strain (LL1111).** Cells were grown in sealed glass bottles in MTC medium with 20 g/L (∼60 mM) cellobiose. In some cases, cells were grown in the presence of 10 g/L (∼220 mM) ethanol. Error bars represent one standard deviation (n ≥ 2). AdhE mutants are grouped by the enzymatic features of the mutation. Within each group they are sorted in amino acid residue order. A complete set of fermentation data is available as Table S7.

Next, we measured the effect of the AdhE mutations on ethanol production (Fig. 3). In the absence of added ethanol, all strains consumed roughly two-thirds of the ∼ 60 mM cellobiose that was initially present in the growth medium. Glucose, lactate, and acetate production were also similar across the strains. The product that showed the most variation was ethanol production. The empty vector control showed no ethanol production (as expected). One group of mutants (P525L, A601E, P628S, and P704L) showed ethanol production similar to that of the WT strain of about 34 mM. Another group of mutants (G553R and H734R) showed less ethanol production, about 20 mM. A third group of mutants (D494G and D494G P525L) showed higher ethanol production of about 60 mM.

In the presence of 10 g/L added ethanol, the fermentation results were very different. One group of strains (WT, P525L, A601E, P628S, and P704L) exhibited substantial inhibition of substrate consumption, consuming only about 15% of the cellobiose initially present. Consequently, these strains produced low levels of fermentation products. In some cases, ethanol production was slightly negative, indicating possible ethanol consumption. The empty vector control consumed about two-thirds of the 60 mM cellobiose initially present, and converted this to lactate and acetate (but not ethanol, as expected, in the no-ethanol condition). The remaining strains (D494G, D494G P525L, G553R, H734R, and P704L H734R) exhibited similar fermentation behavior to the empty vector control, except that they all produced 10 to 20 mM ethanol.

In two cases, we observed multiple AdhE mutations in the same strain, and these mutations were characterized both in combination (as they were found in our evolved strains) or separately. Individually, the D494G mutation improved ethanol production (regardless of the presence or absence of added ethanol). The P525L mutation had little to no effect on fermentation products (compared to WT AdhE), regardless of the presence or absence of added ethanol. When the two mutations were combined, the effect on fermentation products is indistinguishable from that of D494G alone.

The second case of multiple AdhE mutations involved the P704L H734R pair. These mutations were found in a strain of *C. thermocellum* adapted for growth in the presence of 50 g/L added ethanol (10, 12). Individually, the P704L mutation had very little effect on its own. The H734R mutation is more complicated. In the absence of added ethanol, it slightly decreased ethanol production (compared to the WT AdhE), however, in the presence of added ethanol, it allowed both increased substrate consumption and increased ethanol production. Interestingly, when the P704L mutation is combined with the H734R mutation, ethanol production increases, and this effect is observed regardless of the presence or absence of added ethanol.

Since mutations to *adhE* could affect acetaldehyde accumulation, either due to lack of ADH activity, or high reverse ADH activity in the presence of added ethanol, we also measured acetaldehyde. In general, we found very low levels of acetaldehyde (<1 mM), and the concentration of acetaldehyde was not correlated with different types of *adhE* mutations (Fig. 3).

### Enzyme activity of *adhE* mutants

**Figure 4:**
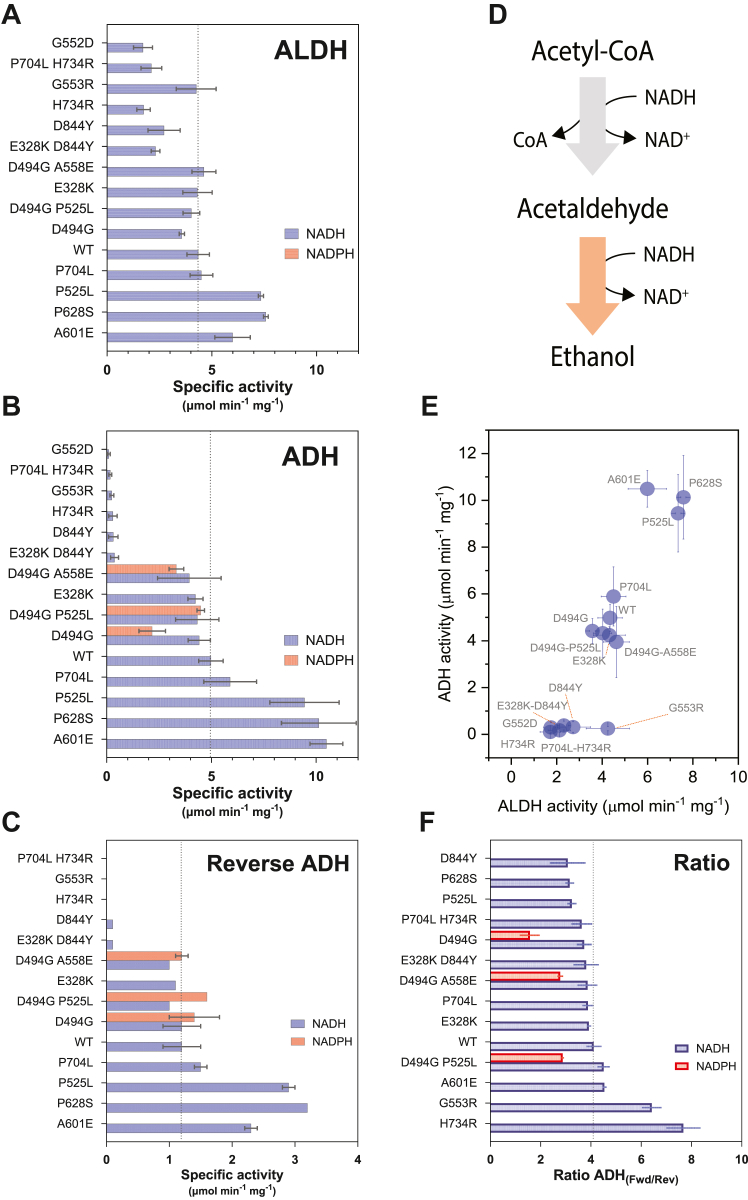
**Comparison of enzyme activity of purified AdhE enzymes.** Enzymes were cloned and expressed in *Escherichia coli*. Purified enzymes were assayed for ALDH and ADH activity with both NADH (*blue bars*) and NADPH (*red bars*) as cofactors (panels *A* and *B*). In cases where no bars are present, that means that activity was below the limit of detection of 0.05 U. Enzymes are sorted by NADH-linked ADH activity. A *vertical dashed line* represents WT activity for each activity. *Panel C* shows reverse ADH activity (*i.e.* ethanol conversion to acetaldehyde), sorted by NADH-linked activity. *Panel D* shows a diagram of the two reactions (ALDH and ADH) mediated by AdhE, in the physiological direction. *Panel E* shows a comparison of ALDH and ADH activity. In this panel, only NADH-linked activity was considered, due to the absence of NADPH-linked activity for the ALDH reaction. *Panel F* shows a comparison of forward and reverse activity for the ADH reaction, sorted by ratio of NADH-linked activity. For all panels, error bars represent one standard deviation, n ≥ 2. ADH, alcohol dehydrogenase; ALDH, aldehyde dehydrogenase.

To better understand how these AdhE mutations affected ethanol tolerance and fermentation behavior, we measured enzyme activity using AdhE enzymes cloned and purified from *Escherichia coli* (Fig. 4). Up to this point, our work was focused on nine AdhE variants. However, as we started to prepare for enzyme assays, we identified additional mutations, and these were also included. In several cases, we unintentionally introduced mutations during cloning (A558E and E328K) and we opted to characterize them, since they were not affected by selective pressure in *C. thermocellum*.

For each enzyme, we measured both ALDH and ADH activity in the forward direction, with both NADH and NADPH cofactors. In addition, we also measured ADH activity in the reverse direction with both NADH and NADPH cofactors (we did not test reversibility of the ALDH domain, since almost all of the mutations were observed in the ADH domain).

The effect of mutation in the ADH domain was relatively clear. One group of mutations significantly reduced activity. This group consists of G553R, H734R, P704L-H734R, D844Y, and E328K-D844Y. Reductions ranged from 91 to 96% depending on the mutation (Table S8). Despite this reduction in activity, small but measurable amounts of activity remained (Fig. S5).

Another group of mutations increased NADH-linked ADH activity. This group consists of P525L, A601E, and P628S.

A final group consists of D494G, D494G-P525L, and D494G-A558E. All of the mutants in this group contain the previously characterized D494G mutation, which increases NADPH-linked ADH activity (17).

Combinations of mutations have interesting effects. By itself, the P525L mutation increases NADH-linked ADH activity. However in combination with D494G, NADPH-linked activity is increased, while NADH-linked activity remains constant. The P704L H734R double mutation combines one mutation that reduces activity (H734R) with one mutation that increases it (P704L). When the mutations are combined, the H734R mutation largely masks the effect of the P704L mutation, and the net result is very low ADH activity.

The effects of mutations on ALDH activity are more difficult to interpret. This is because the ALDH assay produces acetaldehyde, which is a substrate for the subsequent ADH reaction. Thus, if each molecule of acetyl-CoA is converted to acetaldehyde, one molecule of NADH is converted to NAD^+^; however, if the acetyl-CoA is converted to acetaldehyde and then to ethanol, two molecules of NADH are converted to NAD^+^. It is therefore possible that variation in measured ALDH activity is simply an artifact resulting from changes in ADH activity.

We hypothesized that one potential purpose of mutations in the ADH domain could be to bias the reaction direction. To test this, we measured reverse ADH activity for all of our purified mutants. (Note, although it has been previously reported that cell extracts of *C. thermocellum* do not catalyze reverse ADH activity (ethanol oxidation by NAD^+^) (23), our purified AdhE proteins readily perform this conversion). We did not see any effect of mutations on reaction direction. For all the mutations we tested, the magnitude of the forward and reverse reactions were affected to a similar degree (Fig. 4). Two possible exceptions are the G553R and H734R mutations, but in both cases, the overall activity was very low.

### Structural analysis of AdhE mutations

**Figure 5:**
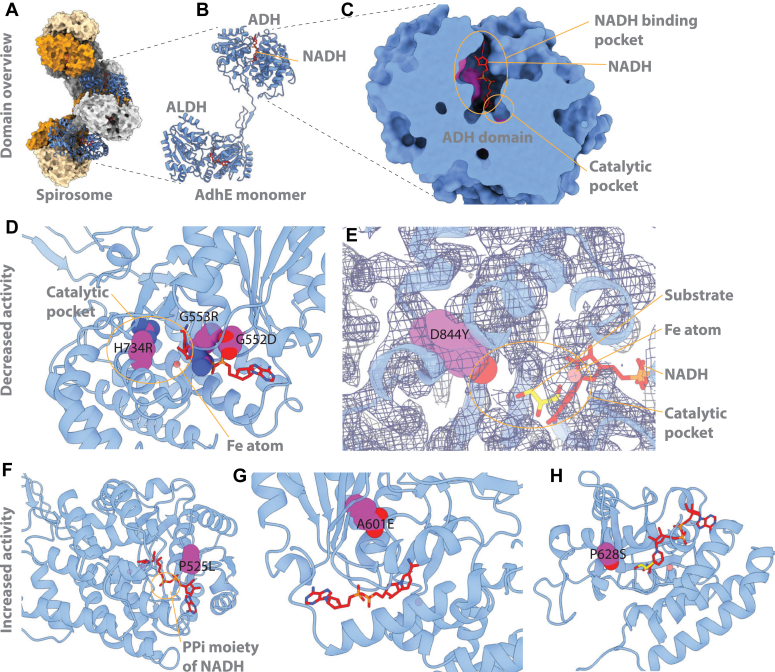
**Structural characterization of mutants.***Panel A* shows the spirosome quaternary structure, composed of multiple AdhE subunits. *Panel B* shows an individual AdhE subunit, which contains both an ADH and ALDH domain. Both domains are shown with bound NAD^+^ (docked from the *Escherichia coli* AdhE structure PDB ID 7BVP). *Panel C* shows a detailed view of the ADH domain with both the NADH binding pocket and catalytic pockets. *Panel D* shows the effect of mutations G552D, G553R, which impinge upon the NADH binding pocket, and H734R, which is in the catalytic pocket. Mutations are shown in *purple*. *Panel E* shows the D844Y mutation, which is also present in the catalytic pocket. Glycerol docked from PDB ID 3ZDR is shown in *yellow sticks* to approximate the acetaldehyde substrate pocket. *Panel F* shows the P525L mutation, which is near the pyrophosphate (PPi) moiety of NAD^+^. *Panel G* shows the buried A601E residue. *Panel H* shows the P628S residue, which is near the catalytic pocket. ADH, alcohol dehydrogenase; ALDH, aldehyde dehydrogenase; PDB, Protein Data Bank.

We used the structure of the extended *C. thermocellum* AdhE spirosome (Protein Data Bank (PDB) ID 8UHW) (24) to examine the effect of the mutants on the active site of the ADH domain (Fig. 5, panels A-C). We observed several consistent patterns. Mutations that reduced activity (G552D, G553R, H734R, and D844Y) were located either in the NADH binding pocket or catalytic pocket (near the Fe atom and nicotinamide ring of NAD(H)) and appeared to interfere with those activities (Fig. 5, panels *D* and *E*). Amino acid residues G552 and G553 are part of a GGG motif that binds to the pyrophosphate moiety of NAD(H) that is well conserved in NADH-binding enzymes (25, 26). The G552D mutation has a longer side chain that reaches into the NADH binding pocket, which results in a reduced space for the NADH molecule, as well as nonideal positioning for the pyrophosphate moiety. Similarly, G553R introduces a bulky side chain that blocks the NADH binding pocket. H734R has the capacity to disrupt both the Fe^2+^ binding site as well as the catalytic pocket, depending on side-chain orientation, and has been proposed to play a role in catalysis—mutations of this residue in similar ADH enzymes have been shown to reduce activity (27, 28). The D844 residue is thought to play a role in binding the acetaldehyde substrate in similar ADH enzymes (29), thus the D844Y mutation may disrupt substrate binding due to the introduction of a bulky side chain into the catalytic pocket (Fig. 5*E*).

For mutants that increase ethanol production without affecting cofactor specificity (P525L, A601E, and P628S), the structural rationale for changes to catalytic activity is less clear. The P525 residue is near the pyrophosphate moiety of the NADH, although outside of the 5 Å radius that is usually considered for bonding. When mutated to leucine, the side chain becomes large enough that it fills the space around the NAD^+^, coming within 3.1 Å of the pyrophosphate moiety, resulting in a tighter, more specific binding pocket (Fig. 5*F*). The P628 residue is near the catalytic pocket, and when mutated to serine, adds the potential of a hydrogen bond in the pocket (Fig. 5H). The A601 residue is buried, and not near the catalytic pocket (Fig. 5*G*). Further, we find that there are no rotamers of the A601 mutant that exist without clashing with the surrounding protein. Therefore, this mutation may affect the larger structure of the protein.

The D494G mutation removes a barrier to NADPH binding in the cofactor binding site described in detail in our previous work (17), the result is that AdhE proteins with this mutation can use either the NADH or NADPH cofactors for the ADH reaction. This was the only mutation that affected cofactor specificity.

The P704L mutation is not located near the active site. Instead, it is located near the interface of two AdhE monomers in the spirosome structure. This could potentially affect spirosome formation, although we did not explore this hypothesis experimentally in this work.

## Discussion

### The effect of AdhE mutations on ethanol tolerance

**Figure 6:**
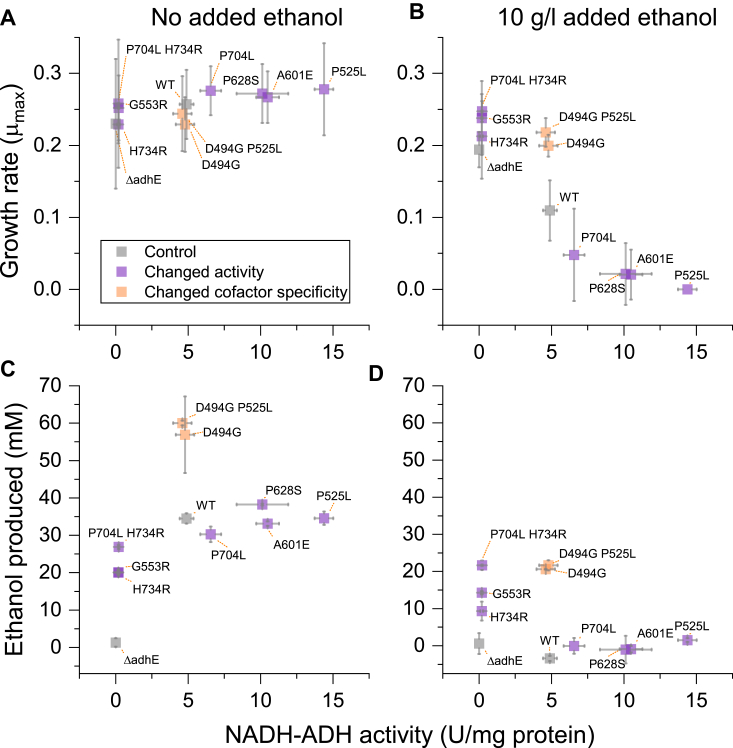
**The effect of AdhE mutations on ethanol tolerance in** ***C. thermocellum*****.** Comparison of the effect of ADH activity *versus* growth (panels *A* and *B*) and ethanol production (panels *C* and *D*) in the presence or absence of 10 g/L added ethanol. AdhE mutations are indicated as labels next to the data point. Error bars represent one standard deviation. For growth rate data, n ≥ 4 biological replicates, for ethanol production and enzyme assay data, n ≥ 2 biological replicates. ADH, alcohol dehydrogenase.

One of our initial goals for this study was to better understand the effect of AdhE mutations on ethanol tolerance in *C. thermocellum*. In the absence of added ethanol, AdhE mutations have no effect on growth rate (Fig. 6, panel *A*). In the presence of added ethanol, however, mutations that decrease NADH-linked ADH activity (G553R and H734R) increase ethanol tolerance. Mutations that increase NADH-linked ADH activity (P525L, A601E, and P628S) decrease ethanol tolerance. This result is consistent with our previous observation that eliminating NADH-linked ADH activity can increase ethanol tolerance (9, 16). We have extended our previous findings by showing that increasing NADH-linked ADH activity reduces ethanol tolerance below that of the WT strain, and larger increases in activity result in larger decreases in ethanol tolerance (Fig. 6*B*).

This result is consistent with a hypothesis we have previously proposed in *C. thermocellum* where high levels of ethanol cause an increase in the NADH/NAD^+^ ratio, which blocks glycolysis at the glyceraldehyde-3-phosphate reaction (30). A similar effect was reported in *Thermoanaerobacter pseudethanolicus* (31). It provides an answer to the question that motivated this work: “why is AdhE a frequent target for point mutations?” The answer appears to be that since AdhE is the sole enzyme providing NADH-linked ADH activity (Fig. S6), and since NADH-linked ADH activity creates extreme ethanol sensitivity (Fig. 2), the cell can increase its ethanol tolerance by mutating the ADH domain of AdhE. This is an important result that informs the design of metabolic pathways for ethanol production in this organism: to avoid a tradeoff between ethanol tolerance and production, ADH activity should be NADPH-linked.

One observation that is not well-explained by this hypothesis is the increase in ethanol tolerance from the D494G mutation. This mutation increases NADPH-linked ADH activity, but does not decrease NADH-linked ADH activity (Fig. 4). It also does not appear to have any effect on acetaldehyde levels (Fig. 3). It is also possible that the increased NADPH-linked ADH activity affects the NADH/NAD^+^ ratio indirectly.

### The effect of AdhE mutations on ethanol production

Another of our initial goals was to understand the effect of AdhE mutations on ethanol production. One surprising result was that the magnitude of ADH activity was almost completely uncorrelated with ethanol production (Fig. 6, panels *C* and *D*). Previously, we have observed a tradeoff between ethanol production and tolerance, where strains that are adapted to tolerate more ethanol produce less of it (9, 20). In the absence of added ethanol, we observe a similar pattern: strains with low levels of ADH activity have reduced ethanol production (Fig. 3); however, in the presence of added ethanol, the reduced-activity mutants (G553R, H734R, and P704L H734R) show more ethanol production. This suggests that ADH activity is in excess under the growth and fermentation conditions tested, and that other factors, such as ethanol tolerance (see discussion above) or cofactor preference (see discussion below), play more significant roles in determining ethanol titer.

An unanswered question in this work is how acetaldehyde is converted to ethanol *in vivo*. Although in the WT AdhE enzyme, this is primarily performed by its ADH domain, several of the AdhE mutants have significantly reduced, although not completely eliminated, ADH activity (Fig. S5). At the same time, even in the complete absence of AdhE, residual ADH activity (NADPH linked) is present in the cytoplasm (Fig. S6). Several genes in *C. thermocellum* have been annotated as putative ADHs, including Clo1313_0076, Clo1313_0166, Clo1313_1798 (*adhE*), Clo1313_1827, Clo1313_1833, and Clo1313_2130 that may be responsible for this activity. Thus, in the mutants with reduced ADH activity, acetaldehyde to ethanol conversion could be due to either thelow levels of residual NADH-linked activity in the AdhE mutants, or the low levels of residual NADPH-linked ADH activity from these other genes. Answering this question will require development of strains where this residual activity has been eliminated, either by deleting genes responsible for putative monofunctional ADH enzymes or by creating AdhE mutants where ADH activity has been completely eliminated.

### Cofactor specificity of AdhE

In contrast to the magnitude of AdhE activity, cofactor specificity played a more significant role in ethanol production. Strains containing the D494G mutation exhibited NADPH-linked ADH activity, and also showed consistent increases in ethanol production relative to the WT enzyme, regardless of the presence or absence of added ethanol (Fig. 6, panels *C* and *D*). The increase in ethanol yield from the D494G mutation may be explained by a stoichiometric linkage with the malate shunt pathway in central metabolism (32, 33, 34). In this pathway, phosphoenolpyruvate is converted to pyruvate *via* oxaloacetate and malate. The net result is a transhydrogenation of NADH to NADPH due to the differing cofactor specificity of the malate dehydrogenase and malic enzyme reactions, and is thought to be one of the main sources for NADPH for biosynthesis in *C. thermocellum* (33). Changing the cofactor specificity of the ADH domain of AdhE (*i.e.* with the D494G mutation) allows this additional electron flux to be diverted to ethanol production, increasing ethanol yield. This stoichiometric explanation is supported by two additional lines of evidence: (1) an increase in ethanol yield in *C. thermocellum* is also observed upon expression of a monofunctional NADPH-linked ADH enzyme from *Thermoanaerobacterium saccharolyticum* (AdhA) (35). (2) disruption of the malate shunt also increases ethanol yield (36), but only in strains with WT (*i.e.* NADH-linked) ADH activity (35).

In this report, we show that the AdhE enzyme of WT *C. thermocellum* is strictly NADH-linked for both the ALDH and ADH reactions. Although all previous reports agree that both activities are primarily NADH-linked, there has been some disagreement about the significance of low levels of NADPH-linked activity previously reported (17). In this work, by using a larger range of protein concentrations, and by more carefully controlling for the spontaneous degradation of NADPH (which is 10× higher than for NADH), we did not observe any NADPH-linked activity for either the ALDH or ADH reactions. These results are consistent with what is known about AdhE enzyme structure (17), and the role of amino acid interactions with the 2′ phosphate moiety of NAD(P)H in determining cofactor specificity (37).

### Substrate channeling

Substrate channeling is a phenomenon where the product of one reaction is directly transferred to the active site of a second reaction without equilibration in the bulk solution (38, 39). Because the AdhE enzyme mediates both ALDH and ADH activity, it has been proposed that the enzyme might also allow substrate channeling between these domains, and a putative substrate channel has been identified (24, 40, 41); however, the extent of substrate channeling (if it even occurs) has not been experimentally determined.

The experiments described in this work provide insight into substrate channeling. If the WT enzyme exhibited 100% substrate channeling (*i.e.* all acetaldehyde molecules generated by the ALDH reaction were transferred directly to the ADH reaction), eliminating the ADH reaction would also eliminate ALDH activity, which would stop after the substrate channel had filled with acetaldehyde molecules (approximately 15–30 molecules). However, this was not observed (Fig. 4), and even in cases where ADH activity was significantly reduced (>95%), ALDH activity was never reduced by more than two-thirds relative to the WT enzyme. The presence of relatively high levels of ALDH activity, even when ADH activity has been reduced (Fig. 4), suggests that at least some of the acetaldehyde generated by the ALDH reaction is released into the bulk solution. In some cases, mutations that significantly reduce ADH activity of AdhE have either a large effect on ALDH activity (H734R) or a small effect (G553R and D844Y). Both of these imply that substrate channeling is at best partial, and may not be present at all. Despite this, acetaldehyde was observed to accumulate only to low levels (2–10% of ethanol concentration) (Fig. 3). An important direction for future study will be to understand the importance of substrate channeling for ethanol production.

## Experimental procedures

### Plasmid and strain construction

Constructs for expression of *adhE* genes in *C. thermocellum* were based on the pDGO143 plasmid we previously developed (18). A previously constructed *adhE* deletion strain (LL1111) (21) was used as the host. AdhE point mutations were introduced using QuikChange mutagenesis (Agilent Technologies) with slight modifications. Plasmids were transformed into *C. thermocellum* as previously described (42). Constructs for expression of His-tagged *adhE* genes in *E. coli* were based on a modified pET-28 vector called pCB17 (24). Routine cloning was performed in *E. coli* NEB5ɑ (C2987, New England Biolabs) (Table 1).

### Ethanol inhibition assays

Growth inhibition by ethanol was measured by growing cells in a 96-well plate with various concentrations of added ethanol, as described previously (9). To detect and correct for evaporation, ethanol concentrations were measured before and after each growth assay using an enzymatic ethanol concentration assay (43).

### Fermentation product analysis

For analysis of fermentation products, *C. thermocellum* cells were grown in MTC-5 chemically defined medium (44). Thiamphenicol at a concentration of 12 μg/ml was added to maintain the plasmid. A 50 ml working volume was used with a 2% inoculum (1 ml). A 1.0 ml fermentation sample was taken from each serum bottle using a 5.0 ml syringe equipped with an 18G needle and transferred to a 2.0 ml Eppendorf tube. The samples were centrifuged for 5 min at 21,300g to pellet the cells. Subsequently, 800 μl of supernatant was transferred to a new Eppendorf tube. A 100 μL aliquot of supernatant was diluted 10-fold in 900 μL of MilliQ water in an Eppendorf tube. The remaining 700 μL of supernatant was stored at 20 °C for subsequent preparation for running on a BioRad Aminex HPX-87H column to quantify fermentation products: cellobiose, glucose, lactate, formate, acetate, and ethanol, as described previously (44).

Acetaldehyde was measured by HPLC by derivatization with 2,4-dinitrophenylhydrazine (45) (https://www.shimadzu.com/an/literature/hplc/jpl214038.html). For measurement of acetaldehyde, the diluted sample was vortexed for 10 s. A 100 μL aliquot of the diluted sample was added to 500 μL of derivatization reagent in a new Eppendorf tube, vortexed for 10 s, and placed on a nutating mixer in a 4.0 C cold room for 12 to 16 h 1. After mixing, the derivatized samples were centrifuged for 5 min at 2000*g* to pellet any residual debris. In addition, 200 μL of supernatant from the derivatized sample was transferred to an HPLC vial and run on a Shimadzu HPLC system equipped with a Shim-Pack XR ODS column. Acetaldehyde could be accurately quantified down to concentrations of 0.01 mM. A detailed protocol is available at protocols.io (10.17504/protocols.io.4r3l2q294l1y/v1) (46).

### Protein purification

For protein expression in *E. coli*, AdhE expression plasmids were transformed into *E. coli* T7 Express lysY/Iq (C3013, New England Biolabs). *E. coli* strains were cultured from frozen glycerol stocks in solid LB medium (Thermo Fisher Scientific) supplemented with appropriate antibiotics. For protein expression, liquid cultures were grown aerobically in Terrific Broth medium (T0918, Sigma-Aldrich) with the appropriate antibiotic to midexponential phase (absorbance at 600 nm = ∼0.5, usually about 2 h) with shaking at 225 rpm at 37 °C.

Once the cultures reached the midexponential phase, 0.2 mM IPTG (Sigma-Aldrich) was added to the culture to induce protein expression and incubated at 16 °C with shaking at 225 rpm for 18 h. Afterward, induced cultures were transferred to serum bottles and purged with nitrogen gas to generate an anaerobic environment for protein expression. Cultures were incubated for a further 3 h with shaking at 225 rpm at 30 °C before harvest.

All the subsequent steps were carried out anaerobically in an anaerobic glove box (Coy Laboratory Products). Cells were harvested by centrifugation (7000*g* for 15 min); the spent culture was discarded while the pellet was washed once with Tris buffer (50 mM, pH 8.3) and stored anaerobically at −80 °C.

Prior to protein purification, the frozen pellet was thawed on ice and resuspended in 1 ml B-PER protein extraction reagent (Thermo Fisher Scientific) with Ready-Lyse Lysozyme and OmniCleave Endonuclease (Biosearch Technologies). Cell lysate was centrifuged at 13,000*g* for 5 min at room temperature (RT) to remove cell debris. The supernatant containing His-tagged protein was applied directly to a Ni-NTA–agarose purification column (His SpinTrap; Cytiva) with a column volume (CV) of 100 μl. Under anaerobic conditions, the column was subject to affinity column purification according to the manufacturer’s protocol. Briefly, the column was first equilibrated with 6 CV of binding buffer (60 mM imidazole) and then cell lysate was applied to the column. Next, the column was washed twice with 6 CV of binding buffer (60 mM imidazole) and thrice with 6 CV of washing buffer (80 mM imidazole). The His-tagged protein was eluted with 2 CV of elution buffer (200 mM imidazole). Analysis of each consecutive 2 CV elution fraction revealed that most of the ADH activity was present in the first three fractions. Therefore, the first three elution fractions (6 CV) were pooled together. Purified enzymes were stored on ice. The purified protein was stored at −20 °C in elution buffer (note that the His-tag was not cleaved off). Enzymes were typically diluted 300-30,000-fold during enzyme assays.

The *E. coli* strain harboring the pCB17 plasmid (WT *adhE* from *C. thermocellum*) was used as a control to measure ADH (forward or reverse) or ALDH activity.

### Enzyme assays

All chemicals were purchased from MilliporeSigma unless otherwise noted. All enzyme assays used a buffer consisting of the following components: 100 mM Tris–HCl buffer, pH 7.5, 0.01 mg/ml bovine serum albumin (Thermo Fisher Scientific, part number 23209), 250 mM NaCl (Thermo Fisher Scientific, catalog number S271), 2 mM MgCl_2_ (Sigma-Aldrich, catalog number M2670), 1 mM dithiothreitol (DTT, Sigma-Aldrich, catalog number 43816), 10 mM sodium ascorbate (Sigma-Aldrich, catalog number11140), and 0.5 mM ammonium ferrous sulfate (Sigma-Aldrich, catalog number 9719).

Several modifications were made to our previous enzyme assay buffer system (17). To accurately measure ALDH and ADH activity, we needed to use very dilute protein preparations (Fig. S1), and bovine serum albumin was added to the buffer to increase the stability of the enzyme in these conditions. It also reduces binding of AdhE protein to the surface of the multiwell plates used for enzyme activity measurements. NaCl was added to increase the ionic strength of the milieu, which improves protein stability (Figs. S1–S3). MgCl_2_ was added to the buffer because in some organisms, we have observed the ALDH activity is activated by MgCl_2_ (35, 47). DTT, sodium ascorbate, and ammonium ferrous sulfate were added to the buffer to reduce metal-catalyzed oxidative damage of key histidine residues in the ADH domain (48) (Figs. S1–S3).

ALDH activity was assayed in the physiological (acetaldehyde forming) direction using the following assay. In addition to the standard buffer mixture (above), the reaction mixture contained: 0.45 mM NADH (Sigma-Aldrich, catalog number N8129) or NADPH (Sigma-Aldrich, catalog number N7505), and 1 mM acetyl-CoA (Sigma-Aldrich, catalog number A2056). The reaction was started by adding acetyl-CoA. The reaction volume was 60 μl. Reactions were performed in a 384-well microplate.

ADH activity was assayed in the physiological (ethanol forming) direction using the following assay. In addition to the standard buffer mixture (above), the reaction mixture contained: 0.45 mM NADH or NADPH, and 10 mM acetaldehyde (Sigma-Aldrich, catalog number 402788). The reaction was started by adding acetaldehyde. The reaction volume was 60 μl. Reactions were performed in a 384-well microplate.

ADH activity was assayed in the reverse (acetaldehyde forming) direction using the following assay. In addition to the standard buffer mixture (above), the reaction mixture contained: 200 mM semicarbazide HCl (Sigma-Aldrich, catalog number S2201), 10 mM NAD^+^ (Sigma-Aldrich, catalog number N6522) or NADP^+^(Sigma-Aldrich, catalog number N5755), and 1000 mM ethanol (Koptec part number V1001). The reaction was started by adding acetyl-CoA. The reaction volume was 60 μl. Reactions were performed in a 384-well microplate. The purpose of the semicarbazide is to react with acetaldehyde, to allow the reaction to proceed further in the acetaldehyde formation direction.

Previously, we performed enzyme assays in a 1 ml reaction volume in a diode-array spectrophotometer. The number of assays being performed in this work required the development of a higher throughput system for measuring enzyme activity, which is why we switched from individual cuvettes to a 384-well microtiter plate. There are several competing constraints that limit the conditions for this assay.

One set of constraints is related to NAD(P)H concentration. It is easier to maintain initial conditions with high concentrations of substrate, but the maximum concentration is limited by the absorbance range of the instrument. We designed our assays to have an initial absorbance of around 2.0 (arbitrary absorbance units) at 340 nm, which corresponded to an NAD(P)H concentration of 0.45 mM, given our pathlength of 0.7 cm (for a 60 μl assay volume in a 384 well microplate).

Another constraint is related to the temperature. Starting the enzyme assay often results in a decrease in temperature of the reaction (a combination of exposure of the plate to RT air, and addition of the RT start substrate). This effect is exacerbated in microtiter plates due to the difficulty of controlling temperature while loading the plates, and the relatively large volume of start reagent (20 μl start reagent added to 40 μl reaction mixture, for a 60 μl final volume). The conventional approach to address this problem is to reduce the enzyme concentration, slowing down the reaction. However, there is a lower limit to the rate at which NAD(P)H-consuming reactions can be measured due to the spontaneous degradation of NAD(P)H, since the signal of the NAD(P)H-consuming reaction is masked by the spontaneous degradation of NAD(P)H. As the temperature is increased, both the time needed for equilibration and the rate of spontaneous degradation increase. Previously we have run our assays at 55 °C, to closely mimic the physiological growth conditions of *C. thermocellum* (17). However at 55 °C, it was not possible to find enzyme concentrations where we could get similar results from our 384-well microtiter plate assay and our 1 ml cuvette assay. Therefore, we reduced the temperature from our assay from 55 °C to 40 °C (as has been done by others studying *C. thermocellum* (23)), and this allowed better agreement between enzyme assays performed in different instruments.

### Automated assay pipetting

Enzyme assays were set up using an Opentrons OT-2 liquid handling robot (Opentrons Labworks Inc) housed in anaerobic glove box (Coy Laboratory Products). First, a two-fold dilution series of the AdhE enzyme was prepared. The initial dilution contained 10 μl of purified protein (0.5–2 mg/ml) in 990 μl buffer (*i.e.* a 1:100 dilution). Next, standard curves were prepared for NADH and NADPH. Assay wells were loaded as follows: First, 20 μl of buffer was pipetted into each well, then 20 μl of enzyme was added (eight two-fold dilutions were used for each enzyme, to ensure that at least two specific activity measurements were within the linear range). At this point, the plate was briefly heated to 50 °C. Then RT start solution (20 μl per well) was added, cooling the plate down to approximately the 40 °C assay temperature. A sealing film (ThermalSeal RTS, Excel Scientific) was applied to prevent evaporation. The plate was then loaded into a prewarmed microplate reader (Agilent BioTek Epoch2), shaken for 30 s, and the absorbance at 340 nm was measured for 3 to 4 h at the minimum interval (∼15 s per read). The BioTek Epoch2 reader is a monochromator-based instrument with a bandwidth of 2.9 nm. The Python script used to run the OT2 robot and associated Excel file with the experimental setup are included as supplementary files (Supplementary Dataset S9).

### Enzyme assay data processing

Using the standard curve, the NAD(P)H concentration was determined from the absorbance data. The rate of spontaneous degradation for NADH and NADPH was determined from the standard curve wells by fitting to an exponential decay function. For assay wells, the first 15 min of data were ignored to allow for thermal equilibration. A sliding 15-min window was then used to determine the rate of reaction. For slope measurements, a minimum R^2^ value of 0.8 was used as a cutoff to eliminate noisy data. For each enzyme dilution, specific activity was determined by subtracting the slope due to spontaneous cofactor degradation, and dividing by the protein concentration. For each assay, the limit of detection was determined by multiplying the NAD(P)H degradation slope by an arbitrary multiplier of three. For each enzyme, the final specific activity was determined based on the average of the two highest dilution assays (*i.e.* most dilute) whose slope was above the limit of detection. Usually, several dilutions gave similar specific activity results. The standard deviation of these technical replicates are reported in the supplemental data (Table S7)

### Protein structural analysis

Protein mutants were examined in the structure of the extended *C. thermocellum* AdhE spirosome (PDB ID 8UHW) (24) using PyMOL (www.pymol.org). Mutants were generated using the mutagenesis tool. The first criterion for rotamer selection was the absence of clashes between the mutant and the surrounding residues. If all rotamers clashed with the surroundings, a secondary criterion was used: minimizing the total strain of clashes. Mutants were then evaluated for whether they affected the ADH NAD-binding pocket, the predicted catalytic site, or outside the area that could affect catalysis.

## Data availability

All of the data are contained within the manuscript or in the supporting information. Strains and plasmids can be obtained upon request.

## Supporting information

This article contains supporting information.

## Conflict of interest

Lee R. Lynd is the co-founder and CEO of the Terragia corporation (https://terragiabiofuel.com/). Terragia has a financial interest in commercialization of *Clostridium thermocellum*. The other authors declare that they have no conflicts of interest with the contents of this article.
