# Supplementary material for: The role of AdhE on ethanol tolerance and production in Clostridium thermocellum
Source: J Biol Chem. 2024 Jul 11;300(8):107559. doi: 10.1016/j.jbc.2024.107559 (PMC11365378; doi:10.1016/j.jbc.2024.107559)
Supplement: Supporting Figures S1–S6 [file mmc3.docx]

# Supplementary Information

# Authors

Angel Pech-Canul ^1,2^, Sarah K. Hammer^1,2^, Samantha J. Ziegler ^2,3^, Yannick J. Bomble ^2,3^, Lee R. Lynd^1,2^, Daniel G. Olson ^1,2,*^

# Affiliations

^1^ Thayer School of Engineering at Dartmouth College, Hanover, NH 03755
^2^ Center for Bioenergy Innovation, Oak Ridge National Laboratory, Oak Ridge, TN, 37830
^3^ Biosciences Center, National Renewable Energy Laboratory, Golden, CO, 80401

* To whom correspondence should be addressed: [daniel.g.olson@dartmouth.edu](mailto:daniel.g.olson@dartmouth.edu)

# Corresponding author information

Daniel G. Olson, Thayer School of Engineering at Dartmouth College, Hanover, NH 03755, daniel.g.olson@dartmouth.edu

**
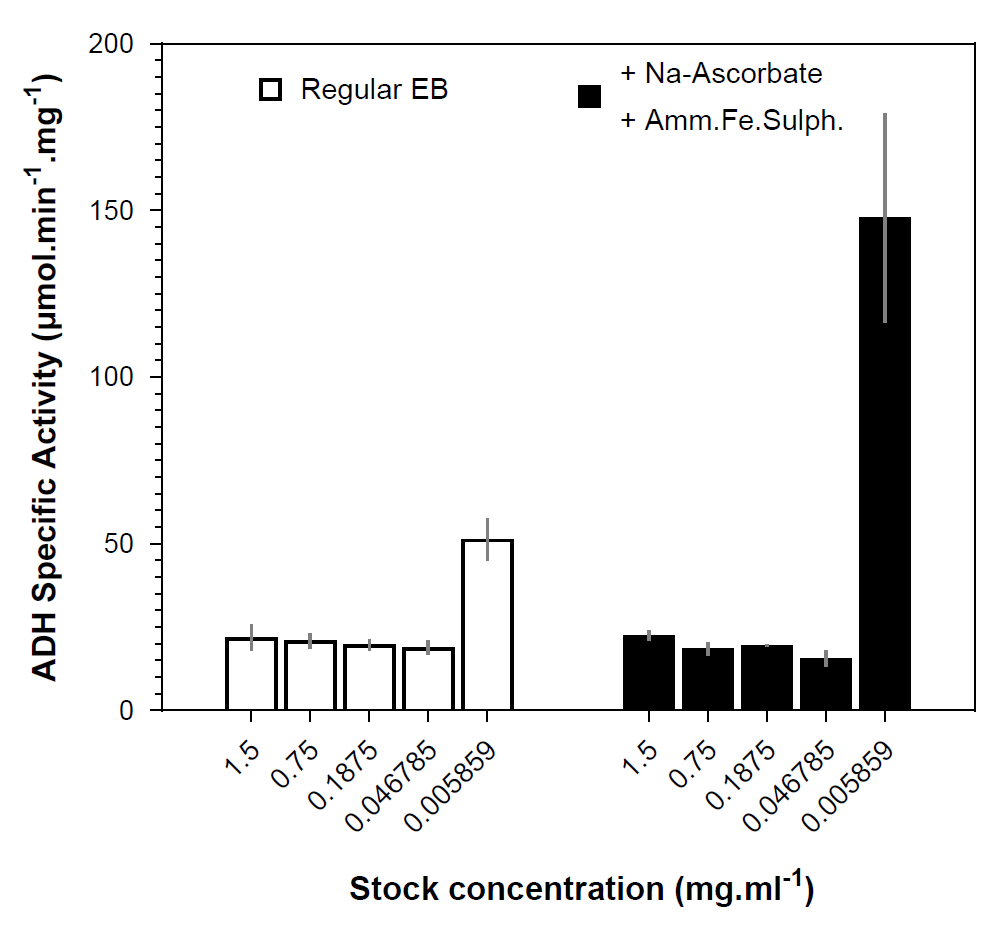
**

**Supplementary Figure S1. Optimizing the forward ADH assay: Assessing the effect of adding reducing agents to the enzyme stock.** A fresh AdhE_C. therm purification prep was carried out as described in “Material and methods” except for the elution step which was performed with either the regular Elution Buffer EB (20 mM Na-Phosphate, 500 mM NaCl, 200 mM Imidazole) or the modified version (EB, 10 mM Sodium Ascorbate, 0.5 mM Ammonium Ferrous Sulphate, 1 mM DTT, 2 mM MgCl_2_). The enzyme stock dilutions were accordingly prepared in their respective EB. The Assay Buffer contained 100 mM Tris-HCl pH 7.5, 0.5 mM DTT, 0.3 mM NADH, purified AdhE, and the reaction was started by adding 20 mM Acetaldehyde. Several stock dilutions of purified AdhE were tested. The assay was run at 55°C. Error bars represent the standard deviation of n ≥ 3 technical replicates.


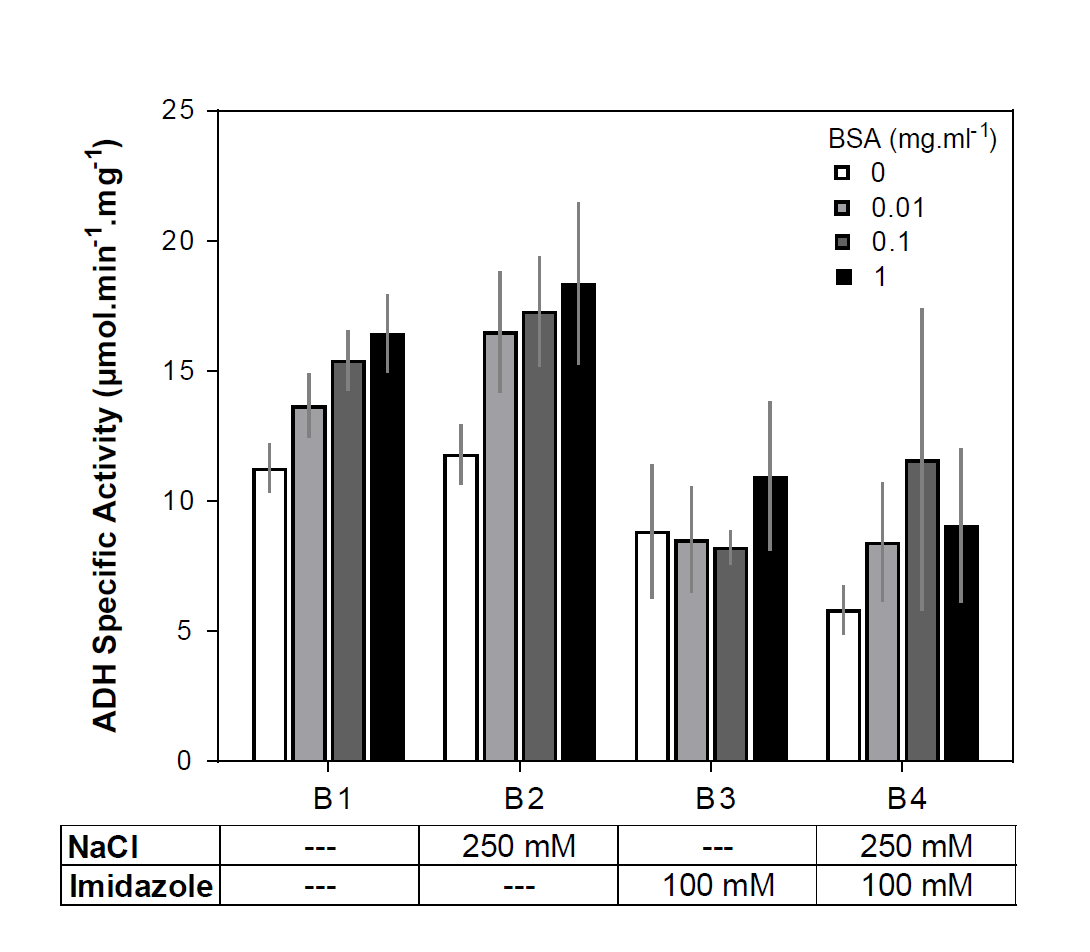


**Supplementary Figure S2. Optimizing the forward ADH assay: Evaluating the effect of adding BSA, NaCl or Imidazole to the Assay Buffer.** Purified AdhE_C. therm was eluted with regular Elution Buffer (20 mM Na-Phosphate, 500 mM NaCl, 200 mM Imidazole) and enzyme stocks were prepared accordingly. The Assay Buffer contained 100 mM Tris-HCl, pH 7.5, 1 mM DTT, 2 mM MgCl_2_, 0.3 mM NADH, purified AdhE, and the reaction was started by adding 20 mM Acetaldehyde. Several concentrations of BSA (0, 0.01, 0.1, 1 mg.ml^-1^) were tested along with the presence/absence of NaCl or Imidazole in the Assay Buffer. The assay was run at 40°C. Error bars represent the standard deviation of n ≥ 3 technical replicates.


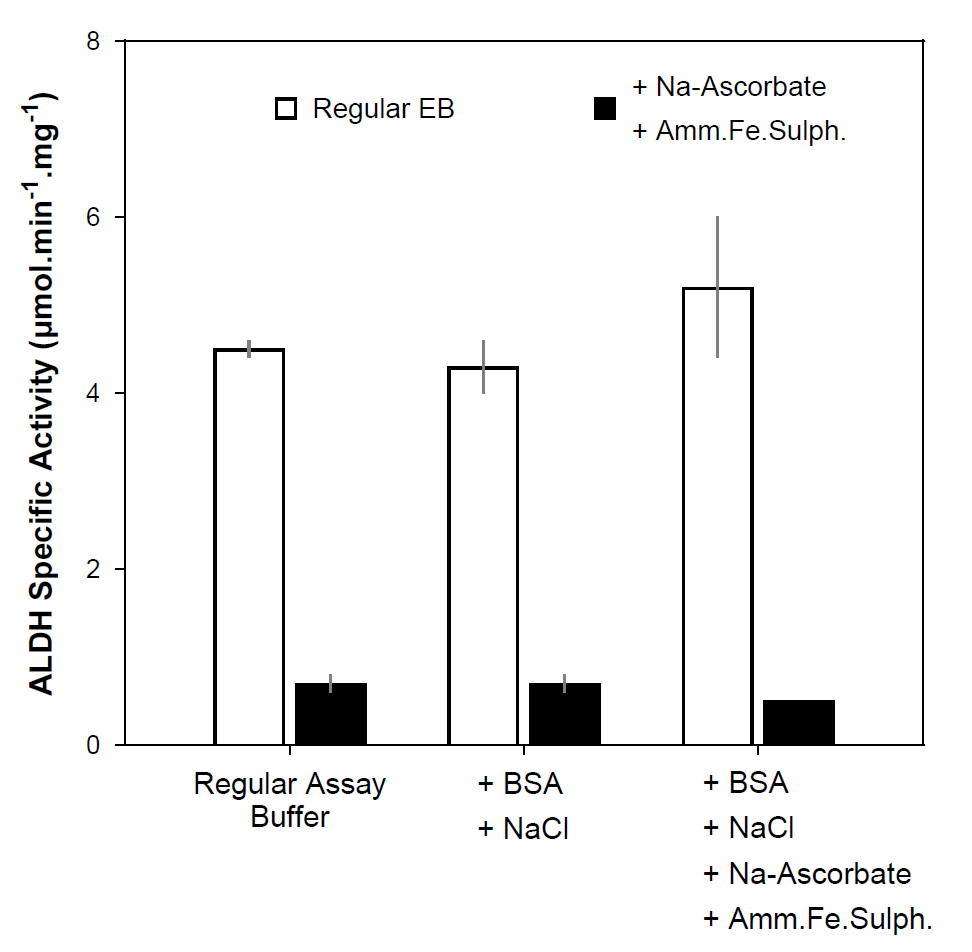


**Supplementary Figure S3. Optimizing the ALDH assay: Evaluating the effect of adding BSA/NaCl and reducing agents to the Assay Buffer.** A fresh AdhE_C. therm purification prep was carried out as described in “Material and methods” except for the elution step which was performed with either the regular Elution Buffer EB (20 mM Na-Phosphate, 500 mM NaCl, 200 mM Imidazole) or the modified version (EB, 10 mM Sodium Ascorbate, 0.5 mM Ammonium Ferrous Sulphate, 1 mM DTT, 2 mM MgCl_2_). The enzyme stock dilutions were accordingly prepared in their respective EB. The Assay Buffer (AB) contained 100 mM Tris-HCl pH 7.5, 0.5 mM DTT, 5 mM MgCl_2_, 0.3 mM NADH, purified AdhE, and the reaction was started by adding 0.3 mM Acetyl-CoA. The ALDH activity was tested using either the Regular AB, AB with BSA/NaCl (0.01 mg.ml^-1^/250 mM, respectively), or AB with BSA/NaCl and reducing agents (0.5 mM Ammonium Ferrous Sulphate, 10 mM Sodium Ascorbate). The assay was run at 40°C. Error bars represent the standard deviation of n ≥ 3 technical replicates.


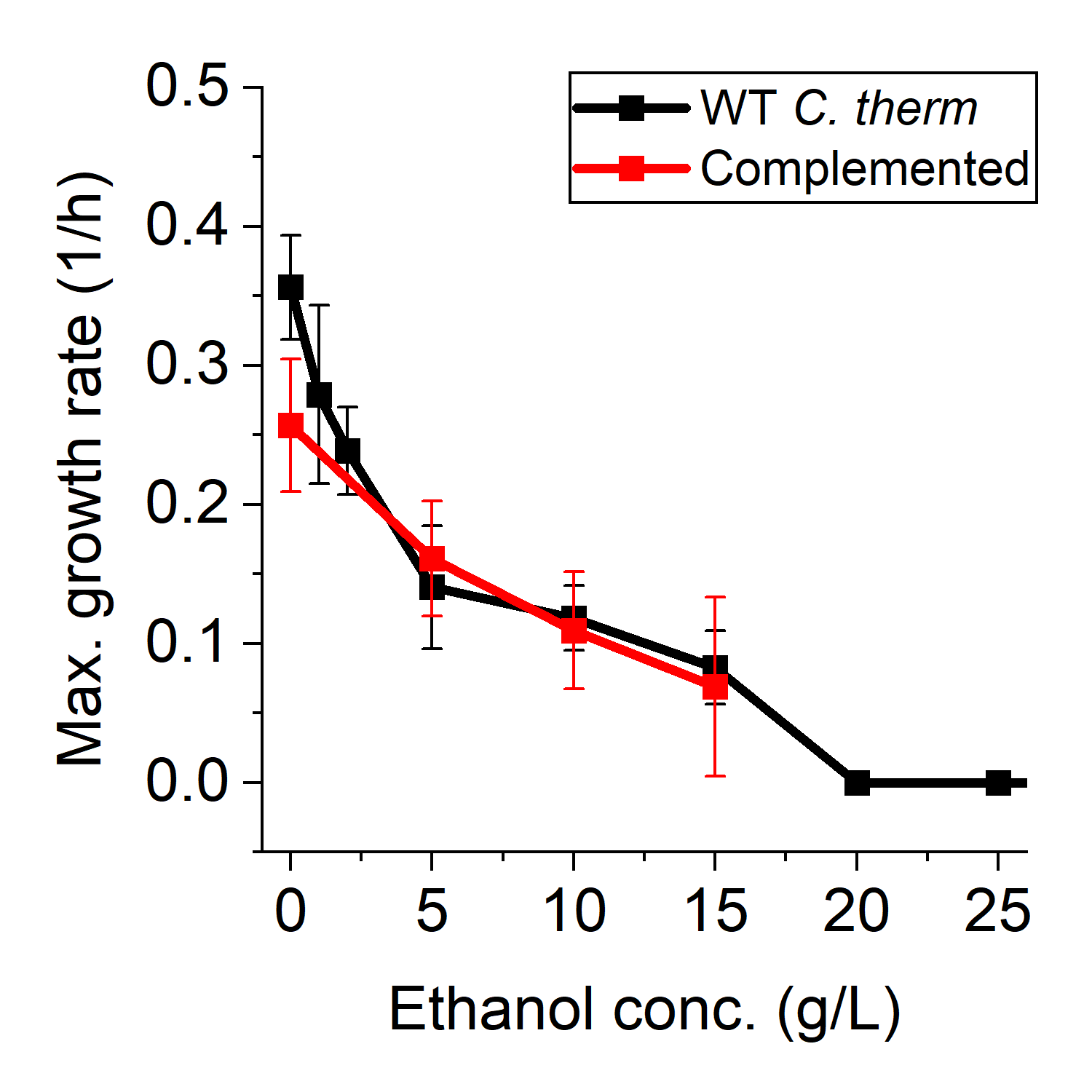


**Supplementary Figure S4.** Comparison between WT *C. thermocellum* (black) and the *adhE* deletion strain complemented by plasmid-based expression of WT *adhE* (red). The data in red is the same data shown in dark gray in Figure 2. Error bars represent one standard deviation, n ≥ 2.


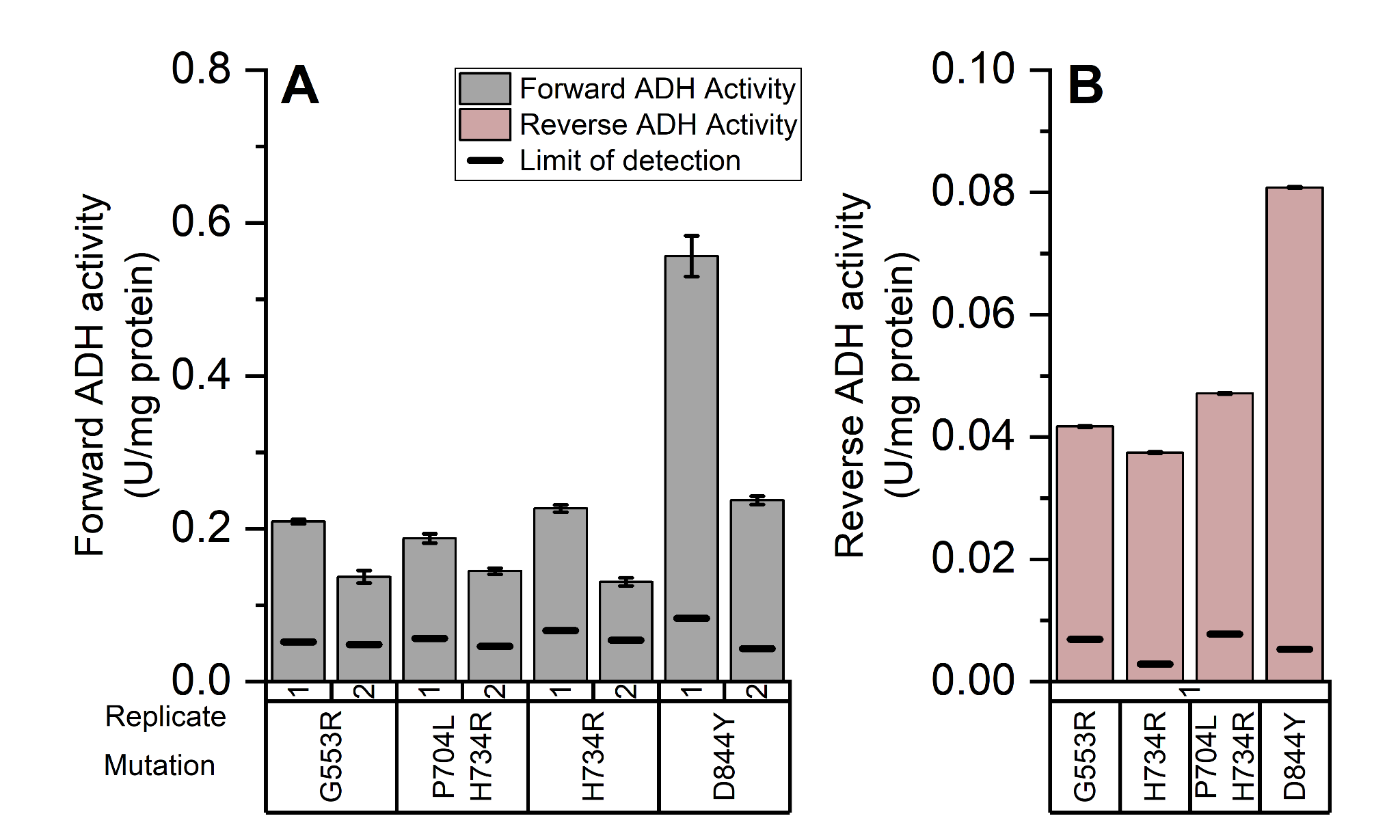


**Supplementary Figure S5.** Residual NADH-linked alcohol dehydrogenase (ADH) activity for AdhE mutants. Analysis of individual replicates shows that residual activity is clearly present in every replicate. Error bars are based on the 95% confidence interval of the assay slope measurement (the rate of NADH appearance or disappearance). The limit of detection is determined as 3x the slope of the control (no substrate) reaction. Limits of detection differ for different mutants due to differences in the protein concentration of the mutant enzyme preparations.


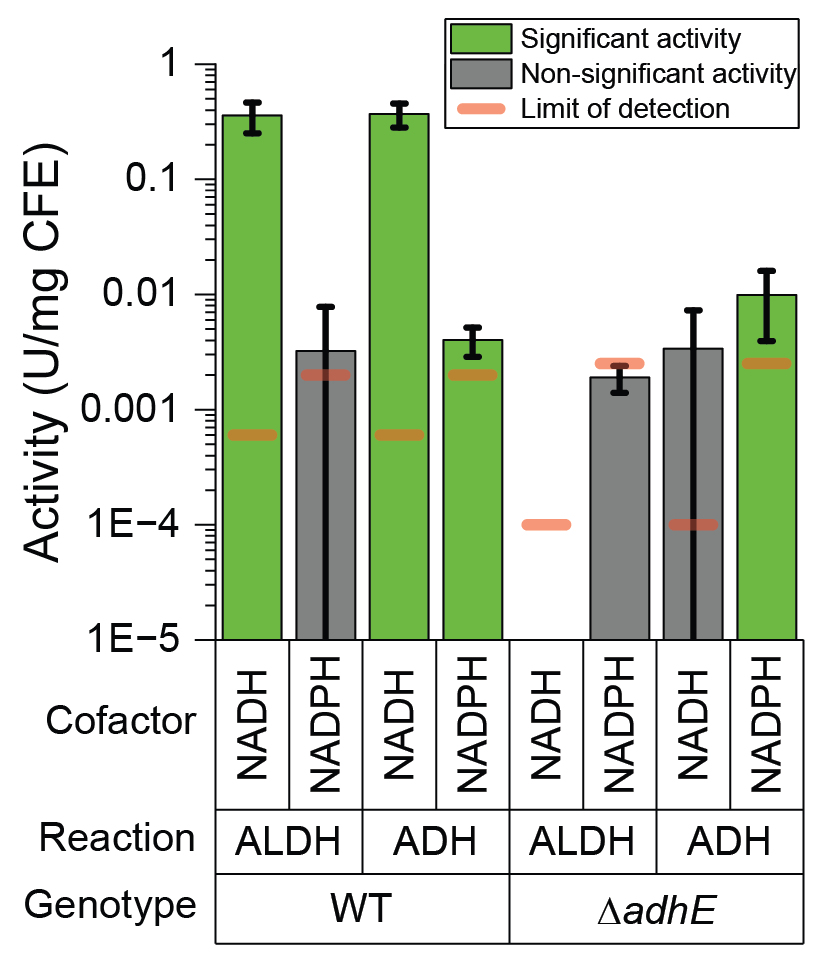


**Supplementary Figure S6**. Residual ALDH and ADH activity in the *adhE* deletion strain of *C. thermocellum*. In WT *C. thermocellum*, ADH activity is primarily NADH-linked (> 99%). In the *adhE* deletion strain, NADH-linked ALDH and ADH activities are eliminated (> 99.9% reduction). The small amount of NADPH-linked ADH activity is not associated with the *adhE* gene, and is not affected by deletion of *adhE*. Error bars represent one standard deviation, n ≥ 2. The limit of detection is set at 3x the value of the rate of spontaneous NAD(P)H degradation at the highest protein concentration tested.

**Supplementary Table S7.** All fermentation data used to generate Figure 3.

**Supplementary Table S8**. Individual replicates for enzyme assay data

**Supplementary Dataset S9**. This is a zip archive that contains files for automated pipetting and subsequent data analysis of robot-assisted enzyme assays.
